# PlanNET: homology-based predicted interactome for multiple planarian transcriptomes

**DOI:** 10.1093/bioinformatics/btx738

**Published:** 2017-11-24

**Authors:** S Castillo-Lara, J F Abril

**Affiliations:** Computational Genomics Laboratory, Genetics, Microbiology and Statistics Department, Institut de Biomedicina (IBUB), Universitat de Barcelona, Barcelona, Catalonia, Spain

## Abstract

**Motivation:**

Planarians are emerging as a model organism to study regeneration in animals. However, the little available data of protein–protein interactions hinders the advances in understanding the mechanisms underlying its regenerating capabilities.

**Results:**

We have developed a protocol to predict protein–protein interactions using sequence homology data and a reference Human interactome. This methodology was applied on 11 *Schmidtea mediterranea* transcriptomic sequence datasets. Then, using Neo4j as our database manager, we developed PlanNET, a web application to explore the multiplicity of networks and the associated sequence annotations. By mapping RNA-seq expression experiments onto the predicted networks, and allowing a transcript-centric exploration of the planarian interactome, we provide researchers with a useful tool to analyse possible pathways and to design new experiments, as well as a reproducible methodology to predict, store, and explore protein interaction networks for non-model organisms.

**Availability and implementation:**

The web application PlanNET is available at https://compgen.bio.ub.edu/PlanNET. The source code used is available at https://compgen.bio.ub.edu/PlanNET/downloads.

**Supplementary information:**

Supplementary data are available at *Bioinformatics* online.

## 1 Introduction

The freshwater planarian *Schmidtea mediterranea*, a platyhelminth of the class *Turbellaria*, has become a model for studying regeneration in animals due to its ability to regenerate its whole body even from small parts of it. Planarians only have one cell type able to divide by mitosis, named neoblasts, which are responsible for the extraordinary regeneration capabilities of these organisms (Wagner *et al.*, 2011).

In recent years, several studies have been performed in order to unravel the molecular mechanisms of planarian regeneration, as well as its regulation (for instance: Cebrià 2007; Fernandez-Taboada *et al.* 2010; Scimone *et al.* 2010). Additionally, different *high-throughput* RNA-seq experiments have been carried out; up to nine of those transcriptomes are publicly available for *S.mediterranea* alone (Abril *et al.*, 2010; Adamidi *et al.*, 2011; Blythe *et al.*, 2010; Galloni, 2012; Kao *et al.*, 2013; Labbé *et al.*, 2012; Resch *et al.*, 2012; Rouhana *et al.*, 2012; Sandmann *et al.*, 2011; Solana *et al.*, 2012), and more datasets are coming for this and related species (Brandl *et al.*, 2016).

Gene or protein expression analyses take into account significant statistical differences between two or more experimental conditions; however, the large amount of collected data and the fact that this data usually refers to specific proteins or transcripts can lead to key functional elements to remain hidden. Approaches based in systems biology can help to unravel the importance of the different proteins in particular functional processes, as to help to identify similarities between different protein interactions networks. Those techniques will pinpoint missing components of the network (relative to networks from different species like humans) that may reveal driver components of planarian-specific processes such as regeneration. Furthermore, it is possible that those approaches will also suggest homologous functional candidates to test in planarians as an *in vivo* model. Cross-referencing pathways information with genome and transcriptome data may also be useful for researchers, facilitating the link to the functional annotation over the sequences and cis-regulatory elements around the genic *loci*.

Instead of studying and analyzing individual genes or proteins, focusing on the environment of such elements where those components play their roles may reveal interesting insights. Molecular medicine based on gene and protein networks has been expanding rapidly, and has shown that most disease-causing genes often work together, either forming protein complexes or participating in the same signalling pathways.

Several approaches have been developed in order to infer protein interactions networks from different sources. Sequence homology can be used to predict interactions that have been conserved between species, and the information about these protein interactions can be transferred from one species to another using different approaches (Garcia-Garcia *et al.*, 2012; Murakami and Mizuguchi, 2014; Schuette *et al.*, 2015). In the context of planarians, Lobo and Levin (2015) developed a method to infer regulatory networks from morphological phenotypes distilled from genetic, surgical, and pharmacological experiments. They built a multi-species protein–protein information retrieval tool (Lobo *et al.*, 2016b), which predicted a gene by using regulatory homologs that has been found to play a role in planarian regeneration (Lobo *et al.*, 2016a).

However, these approaches are limited by the currently available phenotypic data on planarians described in the literature; and, although the amount of data collected for this organism is increasing, other approaches based on high-throughput experiment results and large-scale sequence analyses to predict planarian protein–protein interactions will be very useful to the developmental and regeneration research community.

Linking a predicted planarian interactome with a human network may not only provide a useful tool for researchers in order to associate planarian genes with certain cellular functions, but it may also provide a link between planarian regeneration and human molecular pathways.

## 2 Materials and methods

### 2.1 Summary of the protocol

A protocol based on sequence homology was developed to infer possible *interolog* relationships between proteins of one arbitrary species and human. In this work, we predicted interactions for 11 *S.mediterranea* transcriptomes (Supplementary Fig. S1). The method searched for human homologs to a set of transcripts of the desired species through BLAST searches (Altschul *et al.*, 1990), PFAM domain meta-alignments (Punta *et al.*, 2012), and EggNOG alignments (Huerta-Cepas *et al.*, 2016). Then, a set of features was computed for each possible pair of transcripts, using information from 3did (Mosca *et al.*, 2014), gene ontology (GO; Carbon *et al.* 2009), and a human interactome graph. The protocol was first applied to *Drosophila melanogaster’*s transcript sequences; then a random forest classifier was built using this data.

The program TransPipe was implemented in order to automate the whole procedure, taking as input a FASTA file with the *S.mediterranea* transcripts, a hidden Markov model domain database, a FASTA with human sequences and an EggNOG hidden Markov model database. The program also allows to adjust the *E*-value cutoff for each of the alignment methods independently, as well as providing several plots generated using the R module *ggplot* (Wickham, 2009) to visualize the results. The source code is available from https://compgen.bio.ub.edu/PlanNET/downloads, alongside the install information and the required dependencies. The program is distributed under the free software GNU 2 license.

### 2.2 Datasets

#### 2.2.1 Sequences and hidden markov models

With the aim to have a sequence assigned to each of the HUGO Gene Nomenclature Comittee (HGNC) symbols (Gray *et al.*, 2015), a list of identifiers and synonyms was downloaded from that project website. One set of human sequences was built using three databases: SwissProt (version 2014/09 Wasmuth and Lima, 2016), TrEMBL (version 2014/09), and ENSEMBL (gene build 79, GRCh38.p2, Yates *et al.*, 2016).

The mapping of HGNC identifiers against human sequences was done sequentially. First, priority was given to SwissProt sequences, followed by ENSEMBL and finally TrEMBL sequences. Each sequence was assigned to a specific HGNC symbol using the aforementioned synonyms table, looking for sequences in the next database only if a symbol remained unassigned. This constitued the H-Prot dataset.

The PFAM domains were downloaded from the PFAM site, version 27.0; and the EggNOGs hidden Markov models, animals meNOG version 4.0, from the database website. The *D.melanogaster* transcript sequences to train the random forest classifier were downloaded from FlyBase release r5.56 (Gramates *et al.*, 2017).

We predicted interactions over 11 planarian transcripts datasets: Adamidi (Adamidi *et al.*, 2011), Blythe (Blythe *et al.*, 2010), Consolidated (Kao *et al.*, 2013), GBRNA (*S.mediterranea* mRNA sequences retrieved from GenBank), Dresden (Brandl *et al.*, 2016), Graveley (Resch *et al.*, 2012), Illuminaplus (Sandmann *et al.*, 2011), Newmark (Rouhana *et al.*, 2012), Pearson (Labbé *et al.*, 2012), Smed454 (Abril *et al.*, 2010) and SmedGD (Robb *et al.*, 2015).

#### 2.2.2 Protein–protein interactions

The human protein–protein interactions dataset was retrieved from BioGRID (version 3.4.133, Stark *et al.*, 2006) and STRING (version 10, Von Mering *et al.*, 2003). All the nodes were renamed to HGNC symbols when possible, using the HGNC synonyms table, and when no synonym was found; the node remained as an ENSEMBL protein identifier. This whole human gene/protein network included 26 934 nodes and 794 052 edges.

*D.melanogaster’*s protein–protein interactions were downloaded from DroiD FlyBase curated PPI dataset (version 2015_12, Yu *et al.*, 2008).

### 2.3 Homology prediction

The transcript sequences were aligned to the H-Prot dataset using BLASTX and TBLASTN, with an *E*-value cutoff of 10^−1^^0^ in both cases. From the resulting alignments the *best reciprocal hits* were selected. In order to simplify the whole protocol, we selected the translated longest open reading frame (ORF) for each of all the transcript sequences. These ORF were used for the two following procedures.

The alignment to the EggNOG hidden markov models were performed using *hmmsearch* (Eddy, 1998), with an *E*-value cutoff of 10^−^^10^. We have chosen the subset meNOG (version 4.1), restricting the dataset to only those domains that contained a human protein with an HGNC identifier. The program *hmmsearch* was used in order to annotate the PFAM domains on the transcript sequences, using an *E*-value cutoff of 10^−^^10^ and the hidden markov model database of PFAM-A, release 27.0.

The redundancy of the annotation of the domains over the transcripts was reduced by joining several consecutive domains. The conditions used for that merge were the following:

- Both domains should be equal and consecutive.
- Both domains annotated over the ORF should represent different regions of the domain. In order to decide if this condition was met, the overlap between both annotations had to be <25% of the real length of the PFAM domain.
- The distance between the domains over the ORF had to be equal or less than the real length of the domain that is not annotated over the transcript, plus a 25% of the total length of the domain.

Once the PFAM domains were annotated, each transcript sequence and each protein of the H-Prot dataset was transformed into a meta-sequence where the annotated domains were concatenated, producing a string of domain symbols suitable for a meta-alignment. Those constructs were then aligned using the *Needleman-Wunsch* algorithm, with a *match* value of +30, a *missmatch* value of −30, and a *gap* value of −5. The *match* score was also adjusted to the percentage of the domain annotated on the transcript sequence. Best reciprocal hits were also selected.

The best homologous human protein was selected for each transcript using the following criteria:

- If a protein is a unique best reciprocal hit in the EggNOG alignment, set it as the best homolog for that particular transcript.
- Contrarily, if a unique protein has the largest number of supporting evidences from all the different methods, select it.
- Otherwise, if a unique sequence is the best hit in the EggNOG alignment (lower *E*-value), set it as an homolog.
- Or then, if only a sequence is the best BLAST hit (lower *E*-value), select it.
- Else, select the best scoring hit in the PFAM domain meta-alignment.
- If no condition is met, the contig is discarded.

These decision rules were established because of the EggNOG aligment was set to be more reliable than the others, given that it uses hidden Markov profiles instead of similarity searches, and also given that we had assesed the performance of each method separately.

### 2.4 Prediction of interactions

A set of 19 features was computed for each possible pair of transcripts with at least one human homolog:

- **Path length**. The shortest path between the homologous proteins in the human interactome was computed. If no path was found, a value of −1 was assigned. Self-interactions (those pairs with a shortest path of 0) were removed. In order to speed up the prediction, all the shortest paths between all the human proteins were pre-computed using the python module *graph_tool* (Peixoto, 2014).
- **Domain interaction score**. This score is the number of all the PFAM domain pairs found in the transcripts using hmmsearch (*E*-value ≤ 10^−^^10^) that are annotated as interacting in the 3did database.
- **GO normalized term overlap** (NTO) between the homologous proteins (Mistry and Pavlidis, 2008). This GO similarity measure was chosen because of its simplicity and the speed to compute it compared with other similarity scores. For each pair of transcripts and each of the GO domains (‘molecular function’, ‘cellular component’ and ‘biological process’) all the parents in the GO graph for the annotated terms of the two homologous proteins were retrieved. Then, the overlap of these two sets (normalized over the minimum set) was computed. This feature takes values between 0 (no GO term overlap) and 1 (all the annotated GO terms are the same).
- **Alignment measures**. Several of the alignment measures reported by BLAST, hmmsearch, and the meta-alignment, were used to train the classifier: BLAST and EggNOG *E*-values, BLAST query coverage and PFAM meta-alignment score. Finally, a boolean variable for each of the alignments and each of the three methods was defined. This variable was set to ‘True’ if the transcript-human sequence pairs were best reciprocal hits and ‘False’ otherwise.

To build the random forest classifier, a training set of 11 595 *D.melanogaster* interacting pairs was retrieved from DroiD (Flybase curated dataset), and 853, 023 random pairs filtered against the DroiD pairs constituted the non-interacting protein pairs. All the features were manually discretized into fixed ranges specific to each variable. We used the R module randomForest (version 4.6-10, Liaw and Wiener, 2002), setting the number of trees to 1000 and downsampling the non-interacting pairs so that for building each tree the ratio between non-interacting and interacting pairs was 5:1.

For all the performance validation measures the out-of-bag (OOB) votes reported by the module were used. A cutoff of 0.6 votes was set to decide if a pair is interacting. This cutoff was selected by looking for the value that maximized the F-measure (see Supplementary Fig. S2). In order to reduce the search space of interologs, the program TransPipe only considers those pairs with a *path length* ≤ 2, and removes all the pairs that are not connected on the human interactome (*path length* = −1).

### 2.5 Neo4j database

All the predicted interactomes, as well as the annotations of the different planarian transcripts were stored in a Neo4j database, version 3.1.1 (Robinson *et al.*, 2013). The choice of a graph database instead of a relational database such as MySQL was driven by the nature of the data itself: an interactome can be easily stored as a series of nodes and connections. Traversing the graph can then be done in a very time-efficient way, and operations such as obtaining the transcripts/proteins connected to a given node through an arbitrary number of intermediate connections is trivial.

In addition, having the interactomes stored as a series of nodes and connection not only allows us to perform queries faster, but gives us the ability to use different types of connections that are associated to different meanings. All the homology relationships between planarian contigs and human sequences were also stored as connections between nodes. This allows us to map the predicted interactomes over the human protein–protein interactions. Thanks to this, we are able to search planarian interactions using human protein symbols, as well as comparing subgraphs and pathways across all the different predicted interactomes. The PFAM alignments were also stored in this graph, as well as the GO annotations, giving us the ability to, e.g. look for pathways were the genes involved have a particular GO code or a PFAM domain.

**Fig. 1.**
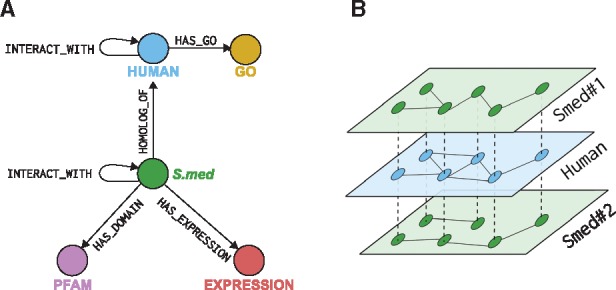
Neo4j database core schema used to store the predicted interactomes, along with the reference human interactome and the sequence annotations for the planarian datasets. **(A)** Diagram summarizing types of relationships and labels used in the database. **(B)** Example of two planarian interactomes (*Smed nos. 1* and *2*) connected through *HOMOLOG_OF* relationships (dotted lines in the figure) to the Human interactome. This database schema allows us to incorporate any number of predicted interactomes in the database, connect them through the Human protein–protein interactions network, and relate similar nodes

Finally, gene expression information from a Digital Gene Expression (DGE) experiment (Rodríguez-esteban *et al.*, 2015) was also stored in the Neo4j database. As can be seen in Figure 1, we used up to five different types of connections, each one with a set of attributes storing the relevant features of that relationship: e.g. *HOMOLOG_OF* relationships have attributes such as the BLAST *E*-value and the sequence alignment coverage.

## 3 Results

### 3.1 Performance of the predictor

**Fig. 2.**
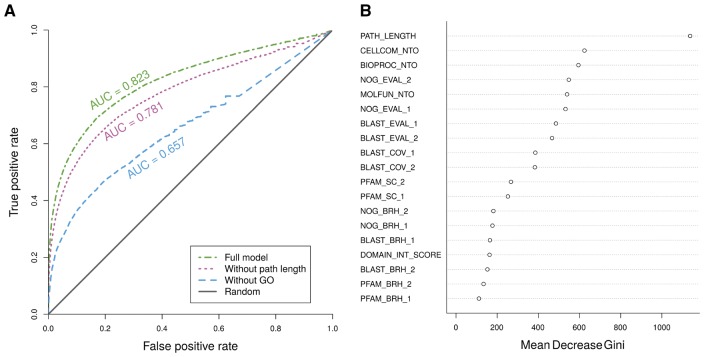
Variable importance and performance of the random forest classifiers used to predict protein–protein interactions. **(A)** ROC curve of the random forest classifiers. The ROC curves were built using the OOB votes of the random forests for the *Drosophila* interacting and non-interacting transcript pairs. We trained three random forest classifiers, one with 19 features (*full model*), another one without the feature ‘PATH_LENGTH’ (*without path length*), and finally, one without the three ‘GO NTO’ features (*without GO*). All of them were compared against a random classifier (solid grey line on the figure). **(B)** Variable importance of the features used by the full model random forest classifier to predict interacting protein pairs. A description of the features can be found in Supplementary Table S1

The performance of the classification of contig pairs as interacting or non-interacting was evaluated using the following measures computed over the out of bag predictions of the classifier: precision, sensitivity, specificity, OOB error rate, and area under the curve of the receiver operating characteristic (ROC); see Figure 2A. The area under the curve calculated using different votes cutoffs was 0.82. In order to improve all the performance measures, but at the same time, to give the user the freedom to choose or focus on more or less confident predictions, we decided to use a votes cut-off of 0.6. By using this cut-off, we obtained a precision of 0.35, a sensitivity of 0.34, a specificity of 0.99 and an OOB error of 2.67%.

We analyzed the relative importance of the 19 features used for the classification of each contig pair using the ‘Gini importance’ index provided by the R randomForest package, as it has been shown to be useful for feature selection in classification problems (Menze *et al.*, 2009). The most useful feature to predict protein–protein interactions resulted to be the distance between the homologous proteins in the human interactome graph, defined as ‘PATH_LENGTH’ in Figure 2B. This is also apparent by the drop in performance seen when removing the feature from the classifier (Fig. 2A). However, when ignoring all three ‘GO NTO’ features, the performance drop is even more pronounced.

### 3.2 Prediction of planarian interactions

The contigs of the 11 planarian transcriptomes were aligned to the H-Prot dataset using BLAST searches, HMMER alignments to EggNOG models, and PFAM meta-alignments. We selected the best hit for each contig and we computed the 19 features for each possible pair of contigs required for the random forest classifier. Although each planarian contig had only one selected homolog, several human proteins had more than one homologous planarian contig in the selected pairs used for the prediction. However, among the selected contig pairs, most human proteins had between one and two homologous contigs for all the datasets (Supplementary Fig. S3). Two human proteins (ACTB and ACTG1) had 6117 and 4222 homologous contigs in the Smed454 dataset, possibly due to the fact that the corresponding RNA sequencing libraries were not normalized. Because of limitations of computing power when predicting the interactions, all except one contig for each of these two human proteins were removed. We selected the contig with the lowest *E*-value in the EggNOG alignment for each of these cases.

The classifier was used to predict interactions in 11 planarian transcriptomes. As it can be seen in Table 1, the number of contigs with a homolog varies from 2314 to 20 665, while the number of human homologs for each dataset shows a way lower variation. This fact leads to some datasets having more unique relationships between planarian contigs and human proteins, and others containing more contigs that align to the same proteins. This is illustrated in Supplementary Figure S3 where the distributions of number of homologs by sequence among transcriptomes can be easily compared. The final number of predicted interactions is also highly variable. However, the number of interactions strongly correlates with the initial number of contigs with an homolog in each dataset (*Spearman*’*s rho* = 0.945, *P*-value < 10^−^^10^). The number of contigs in each predicted interactome is also dependant on the initial contig count (*Spearman’s rho* = 0.873, *P*-value = 9.5 × 10^−^^4^). The overlap between the 11 predicted networks and the human reference interactome is shown in Supplementary Figures S4 and S5. The figures were made using the R package UpSetR (Conway *et al.*, 2017).

**Table 1.** Results of the prediction of protein–protein interactions for 11 *S.mediterranea* transcriptome datasets

Transcriptome	Total contigs	Contigs with homolog	Human homologs	Contigs in interactome	Number of interactions	Average degree	Percentage of Plen1
Adamidi	18 547	9478	5187	4903	32 626	6.657	36.8%
Blythe	24 008	10 930	5564	5929	32 892	5.548	34.7%
Consolidated	23 545	12 775	5809	7098	53 609	7.553	30.8%
Dresden	40 480	14 626	5889	7713	68 805	8.921	30.4%
GBRNA	4675	2314	1547	983	3158	3.213	55.5%
Graveley	19 503	8475	4329	3796	14 254	3.755	30.6%
Illuminaplus	28 926	10 090	5182	5263	29 574	5.619	36.8%
Newmark	53 898	20 665	6359	11 188	100 138	8.950	39.0%
Pearson	25 889	10 465	5656	5176	30 538	5.810	31.0%
Smed454	46 602	14 720	4711	8734	112 512	4.061	57.4%
SmedGD	32 615	12 904	5947	6715	55 939	8.330	28.4%
Mean		11 510	5107	6136	48 550	6.220	
SD		4587	1317	2670	34 151	2.048	

*Note:* The ‘*Average degree’* describes the connectivity of each graph as *interactions*/*nodes*. The ‘*Percentage of Plen1’* corresponds to the fraction of interactions in each network that are also found in the reference human interactome.

In order to compare the confidence of each prediction, we plotted the distribution of votes of the classifier for each dataset (including the OOB votes for the testing dataset). As can be seen in Supplementary Figure S6, all the planarian interactomes have a very similar distribution of votes, with the votes for the testing dataset being slightly higher. Most predictions fall between 60% of votes and 70% of votes, but there is a big number of predicted interacting pairs with a high percentage of votes in all the datasets. For all the datasets, the proportion of contig pairs with interacting homologs in human (*pathlength* = 1) was <1%, while this proportion increased significantly when considering only the available predicted interactions (Table 1). Additional information about both the predictions and the sequence alignments for each dataset is available at the protocol summary page (https://compgen.bio.ub.edu/PlanNET/datasets).

### 3.3 PlanNET web application

To explore the predicted interactomes and the sequence annotations of the planarian sequences, we implemented a web application called PlanNET using the python web development framework Django and the javascript plugin cytoscape.js (Franz *et al.*, 2016). The starting form is divided in four sections that serve as different entry points to the Neo4j database.

GeneSearch provides a text-based search by keywords, thanks to it; the user can look for all the annotated features of the planarian contigs using either planarian contig identifiers or human protein symbols. The latter will retrieve all the *S.mediterranea* contigs of a particular dataset that are homologous to the specified human protein.

We also provide a way to explore the predicted interaction networks utilizing cytoscape.js in *NetExplorer*, where the user can search for nodes across the different planarian protein–protein interaction networks, either using contig identifiers, human protein symbols (wildcards allowed), PFAM identifiers, KEGG pathway identifiers, or GO codes. In Supplementary Figure S7, we can find an example of a human protein network (MAPK signalling pathway, with *KEGG: hsa04010*) projected over the *Smed454* transcriptome.

Thanks to the graph-based database manager Neo4j, traversing the networks to retrieve any subset of them does not have a huge performance impact; we took advantage of this capability to implement *PathwayFinder*. This application looks for all the possible paths between two protein/contigs in the specified interactome, rating all these paths depending on their overall confidence. This score was defined as the mean of the random forest votes for each of its predicted interactions. Just like in *NetExplorer*, users can search by human protein symbols, PFAM identifiers, contig identifiers, KEGG accessions and GO codes.

For the sake of completeness, we also implemented a BLAST web form to look for contigs on the graphs using sequence homology searches.

## 4 Discussion

In this work, we introduce a tool to predict protein–protein interactions from transcript sequences using sequence alignments and a reference Human interactome. This tool was then used to predict 11 different protein interactions networks from 11 *S.mediterranea* transcript datasets. As a result, we provide PlanNET, a web application that allows researchers to explore these networks in different ways, as well as to access to all sequence annotations performed in order to predict the interactions.

Given the OOB performance evaluation of the predictor, we conclude that this random forest classifier is useful for inferring interologies between two species, for instance planarian and human. The area under the ROC curve of 0.82 strongly indicates the significant improvement from a random predictor of our tool. The performance of the random forest classifier is similar to a previously described interaction predictor developed by (Garcia-Garcia *et al.*, 2012) (see Supplementary Table S2). The low precision and sensitivity (0.35 and 0.34, respectively) can be attributed to the fact that from all the possible pairs of proteins of a given organism, only a tiny subset of them really interacts. It has been described that the protein interaction network of any given species is always very sparse, as the degree distribution of most of them follow the power-law (Barabási and Oltvai, 2004). This fact alone makes it harsh for any predictor to retrieve a large amount of interactions out of those pairs, without retrieving many false positives. However, the developed predictor can be further improved in different ways; e.g. introducing new features such as the confidence of the annotated interactions in the reference human network, or adding new reference interactomes from other species.

From all the features used by the classifier, the most important one is the distance of the homologous human genes in the Human protein–protein network (‘PATH_LENGTH’). It is worth to note that, even without the most useful feature, the resulting classifier still shows good performance, with an AUC of 0.781, highlighting the robustness of the other features when trying to predict protein–protein interactions. The GO similarities considered together contribute to the performance of the predictor more than the feature ‘PATH_LENGTH’ alone; and the EggNOG alignment *E*-values are next in importance. The GO similarities and the EggNOG *E*-values paired together, ensure that both proteins have a high sequence similarity to their respective human homologs, that those homologs are known to be in a similar cellular location (*cellular component*) and that they share GO *biological process* and *molecular function* annotations. Thus, our tool not only predicts interactions between putative proteins translated from transcripts, but it essentially clusters these contigs according to their functional similarities (instead of, e.g. their location in the genome). The relative importance of the EggNOG *E*-values may be due to two reasons: first, the performed protocol favors EggNOG alignments when selecting best hits for each contig, and secondly, hidden markov models are known to detect more distant homologies than sequence searches such as BLAST (Park *et al.*, 1998).

The different number of interactions predicted for each dataset can be attributed to the different number of homologs found for each one, as the strong correlation between the ‘*Contigs with an homolog*’ and ‘*Number of interactions*’ suggests. Therefore, the different level of fragmentation of the contigs, apparent from the dissimilarities between homologous proteins found for each dataset (Supplementary Fig. S3), could greatly affect the final result of the prediction. This reinforces the importance of building a proper full-length mRNA reference set, especially for *S.mediterranea*, as it has been shown for model organisms like human (Cho *et al.*, 2014).

**Fig. 3.**
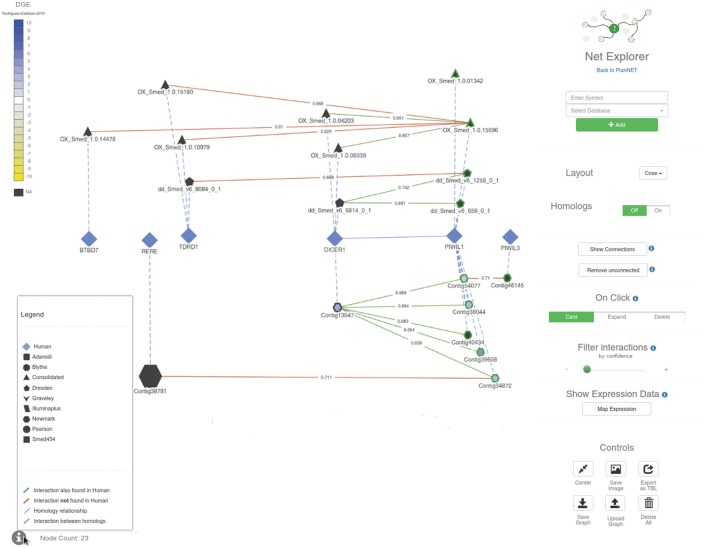
Screenshot of the PlanNET NetExplorer browser. Each node in the graph represents a protein/contig, the shape of the nodes determines the dataset to which they belong. The size of the nodes depends on the node degree (total number of interactions). The edges color varies depending on the type of relationship (see ‘Legend’ on the lower left corner of this figure). DGE data comparing two samples from the experiment described in (Rodríguez-esteban *et al.*, 2015) was projected over this visualization; the color of nodes is based on the expression fold change (scale shown on the upper left corner of this figure). The controls on the right panel allow users to explore the graph further by clicking on nodes, as well as getting information for each contig/protein. Numbers on the edges correspond to the proportion of votes of the random forest classifier, as a measure of the confidence of any given interaction. Users can filter these interactions by confidence value with the slider on the right (under ‘Filter interactions’). Finally, the interface allows researchers to save and load graphs in JSON format (Color version of this figure is available at *Bioinformatics* online.)

Using Neo4j as our database backend, we developed a web application, called PlanNET, to explore not only the predicted networks, but the sequence annotations as well. Apart from BLAST searches and simple text searches (provided by the applications *BLAST* and *GeneSearch* at PlanNET, respectively), we have also implemented two additional ways to explore the protein networks. *NetExplorer* (Fig. 3), will allow researchers to look for predicted interaction networks using contig IDs, KEGG pathway identifiers, GO codes or PFAM domains as baits. The asynchronous javascript searches will facilitate users to dinamically compare the networks annotated for the different transcriptomes with respect to the reference interactome, human in this case. The application *PathwayFinder* provides a simple way to look for protein interaction pathways in the predicted networks; specifying a *starting protein*, an *ending protein*, as well as the length of the desired pathways.

We can also integrate gene expression data from different sources, which has been tested by showing results from a DGE experiment performed by Rodriguez-Esteban *et al.* (2015). This data can be projected over the graphs, coloring the nodes according to the expression levels; right know, the application allows to color the nodes using expression data from one sample (binning colors by percentils of absolute expression levels) or to compare two samples at the same time from the same experiment [assigning colors as a function of *log*2(*FoldChange*)]. We are planning to add further RNA-seq expression data to this tool, which will allow more complex queries to be performed, such as retrieving subnetworks having correlated expression levels across different experiments and samples. Neo4j manager and the Cypher query language make those complex queries simple and fast (Yoon *et al.*, 2017), and they allow to perform them in real time as opposed to pre-computing them.

When designing experiments with the aim to unravel the underlying mechanisms of planarian regeneration, the biological context of any given candidate gene is just as important as its annotations. Thus, a predicted protein–protein interaction network for many planarian transcriptomes will be useful in determining that context from a transcript-centric point of view. Our applications allow researchers to compare the human homologs found in all the transcriptomes, to look for possible interacting proteins, to retrieve sub-networks using GO codes and PFAM domains, and to compare expression levels across the transcriptomes. Our approach focuses on the planarian contigs instead of the annotated genes on a reference genome; giving researchers the flexibility to work with any contig as a proper separate entity with its own annotations. When new refined transcriptomes will be available in the future they will be easily incorporated to PlanNET using our current pipeline, without interfering with all the previous datasets and maintaining the relevance of the application.

At last, in this work we provide one of the first practical applications of the database manager Neo4j to store and analyze multiple protein–protein interaction networks. Sequence homologies predicted for the planarian contigs allow us to link the *S.mediterranea* and the human interactome in the database, making quick queries to compare and traverse the networks easy. Our current database design will facilitate the inclusion of genetic interactions, as well as the extension with new interfaces to explore them in the future. In conclusion, this database engine provides a very adaptable framework for storing, modelling and visualizing many networks projected over a reference interactome. This has been crucial to implement a responsive interactive interface, PlanNET, over the extensible interologs network that projects planarian transcriptomes towards human sequences and vice versa. The analysis pipeline can be applied to any species transcriptomic datasets to map it over any model organism reference interactome, which makes our protocol extensible to a broader range of similar research problems.

## Supplementary Material

Supplementary Data
