# Supplementary material for: PlanNET: homology-based predicted interactome for multiple planarian transcriptomes
Source: Bioinformatics. 2017 Nov 24;34(6):1016–23. doi: 10.1093/bioinformatics/btx738 (PMC5860622; doi:10.1093/bioinformatics/btx738)
Supplement: Supplementary Data [file btx738_supp.zip › btx738-suppl_data/supplementary_materials.pdf]

Supplementary materials

PlanNET: Homology-based predicted interactome for multiple planarian transcriptomes

S. Castillo-Lara and J.F. Abril

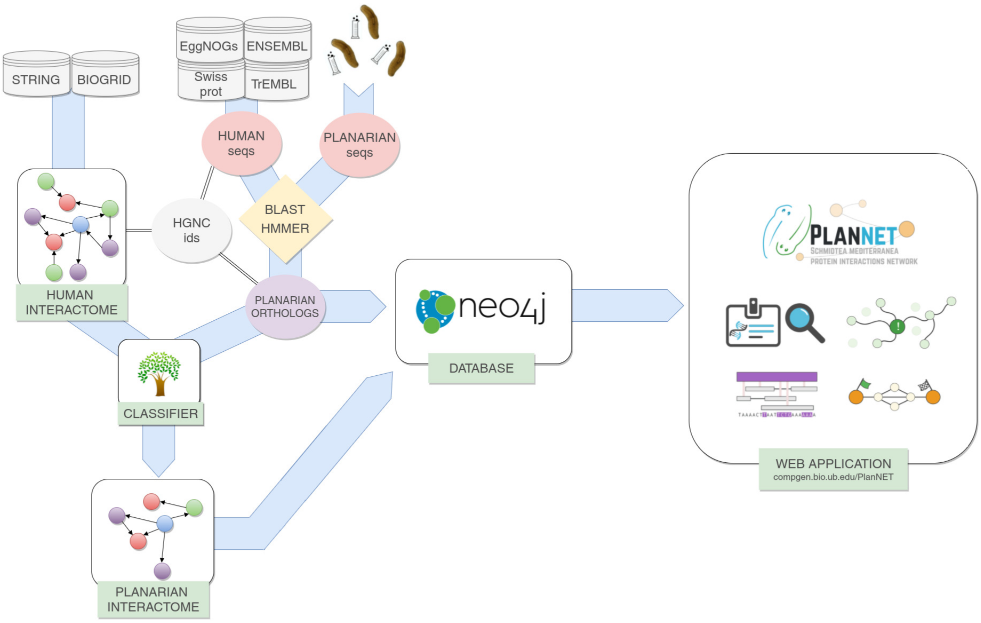

Supplementary Figure 1

General overview of the protocol used to predict protein-protein interactions on *S. mediterranea* transcriptomes. Using a human protein-protein interactions network retrieved from BioGRID and String, we predicted protein interactions for planarian transcripts. First, we searched for homology relationships between human proteins and planarian transcripts using BLAST and HMMER, and then, using a random forest classifier trained with *Drosophila melanogaster* sequences, we predicted a different interactome over each transcript set. All the information gathered during this process was uploaded to a Neo4j database, and we built a web interface called PlanNET to navigate through those networks and explore the connectivity and the nodes content such as sequence and domain information or projected expression levels.

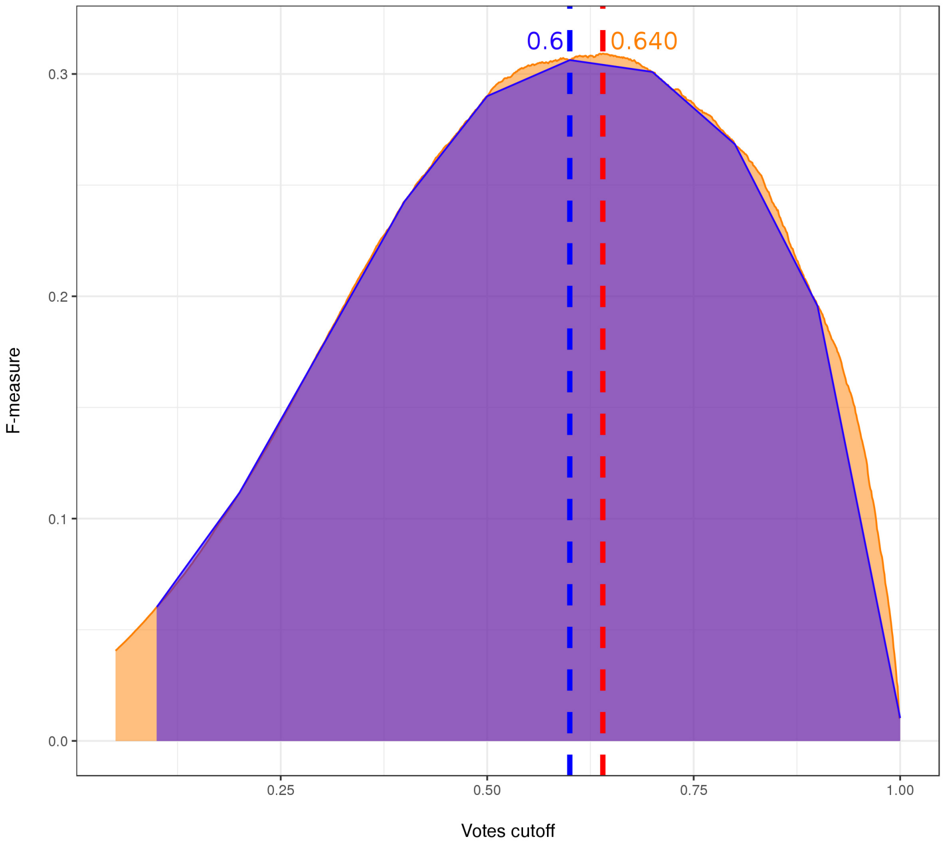

Supplementary Figure 2

F-measure of the full model classifier for different cut-offs of votes. In purple, we have the F-measure for the performed cut-offs for the article, which result in a maximum value for the F-measure of 0.306 on a votes cut-off of 0.6. We have tried the same approach but by increasing the resolution; instead of choosing jumps of votes of 0.1 we have computed the F-measure for all the cut-offs from 0 to 1 in 0.001 increments. This approach results in a maximum F-measure of 0.309 on a votes cut-off of 0.640 (orange on the figure). As we can see in this figure, the difference in the resulting votes cut-off that maximizes the F-measure will not have a great impact on the final performance of the classifier. Because we let the user of PlanNET to filter the interactions by this same cut-off with a slider on NetExplorer, we think that the initial cut-off of 0.6 is good enough for the predictor of protein-protein interactions.

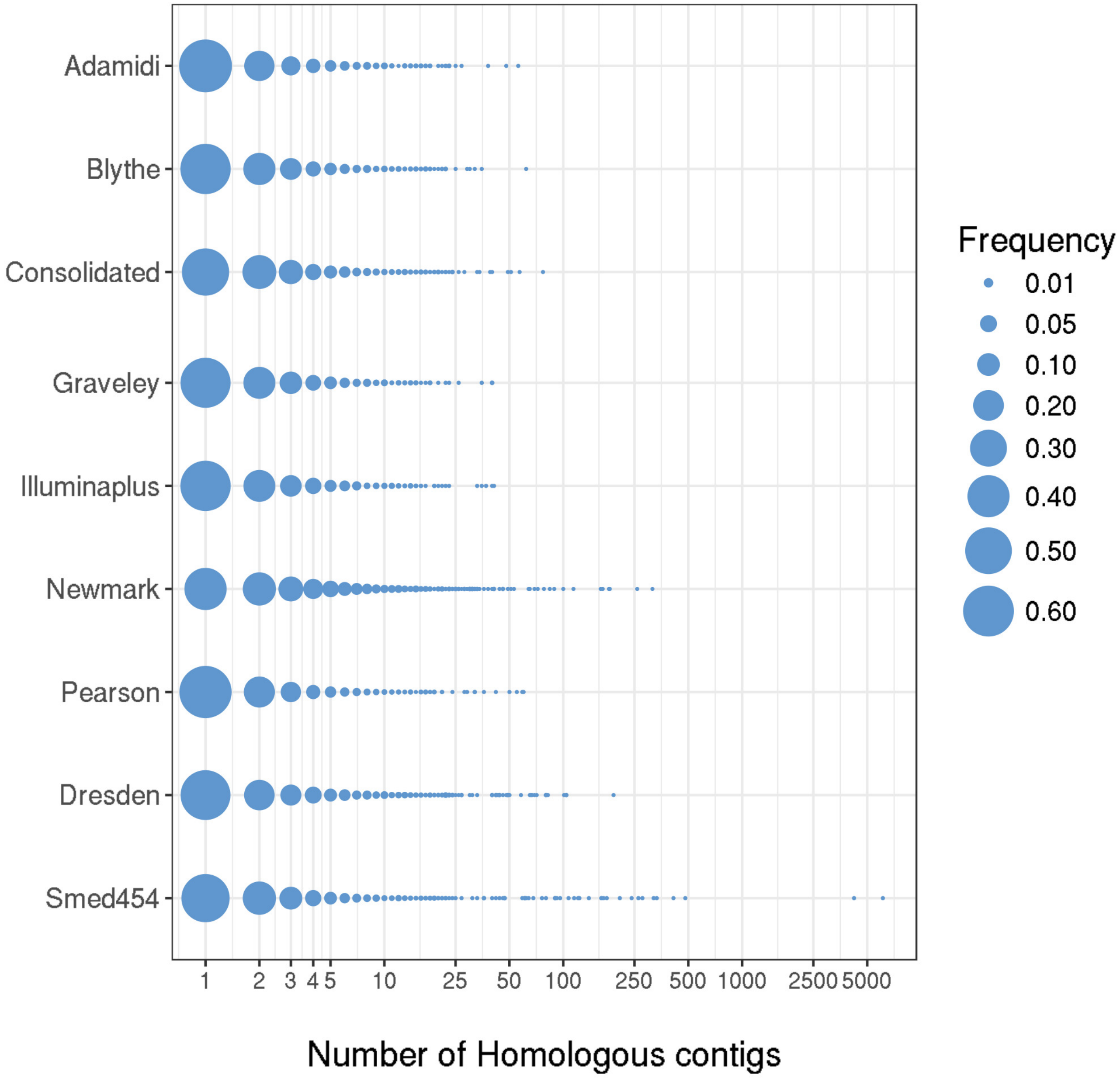

Supplementary Figure 3
Number of homologous contigs for each human protein with at least one homolog in the analyzed transcriptomes.

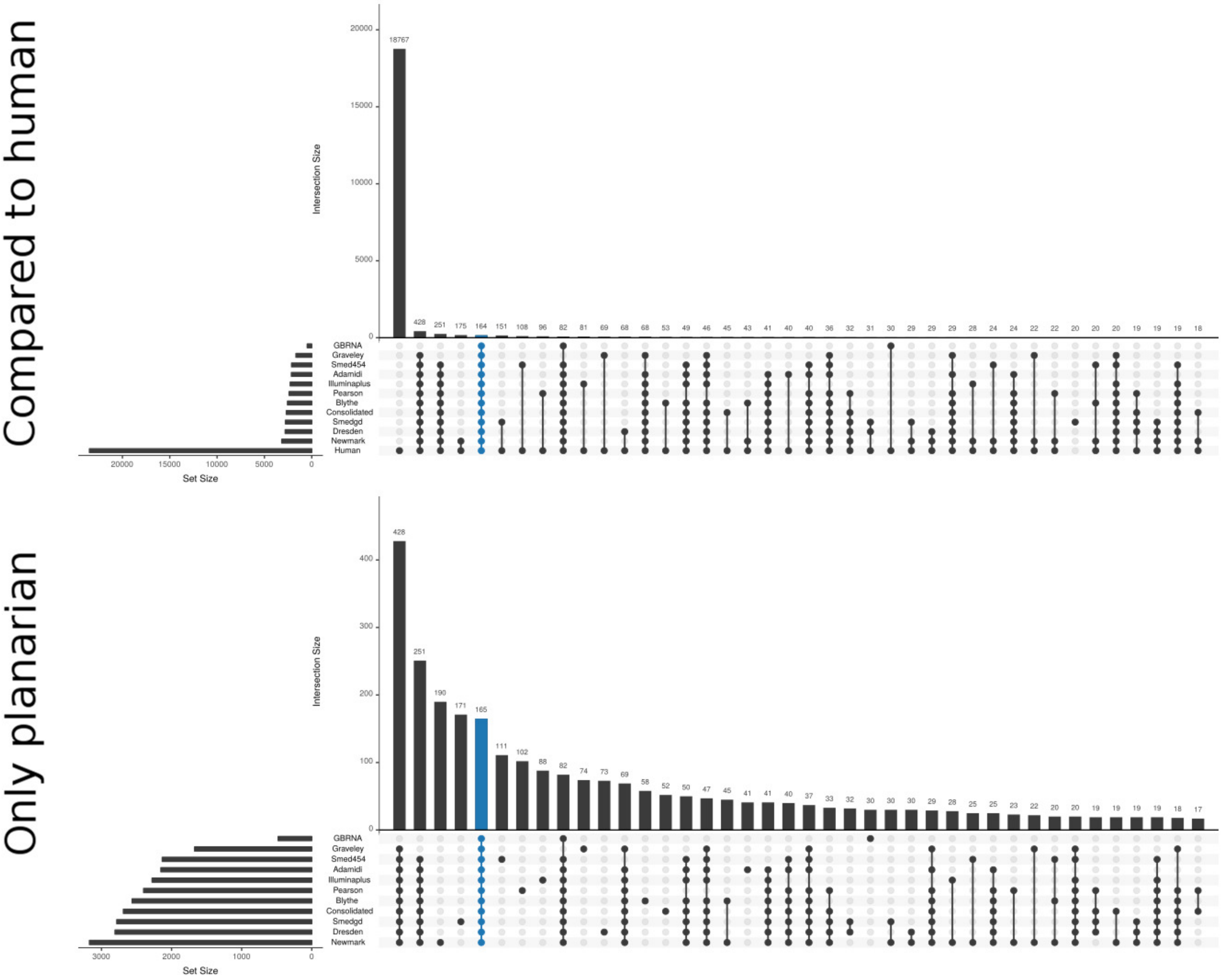

Supplementary Figure 4

Intersection of human protein symbols found in the reference interactome and the predicted interactomes. For the predicted protein networks all the contig pairs were converted to their human homologous protein symbols before the intersections were made. The plots were created using the R package UpSetR.

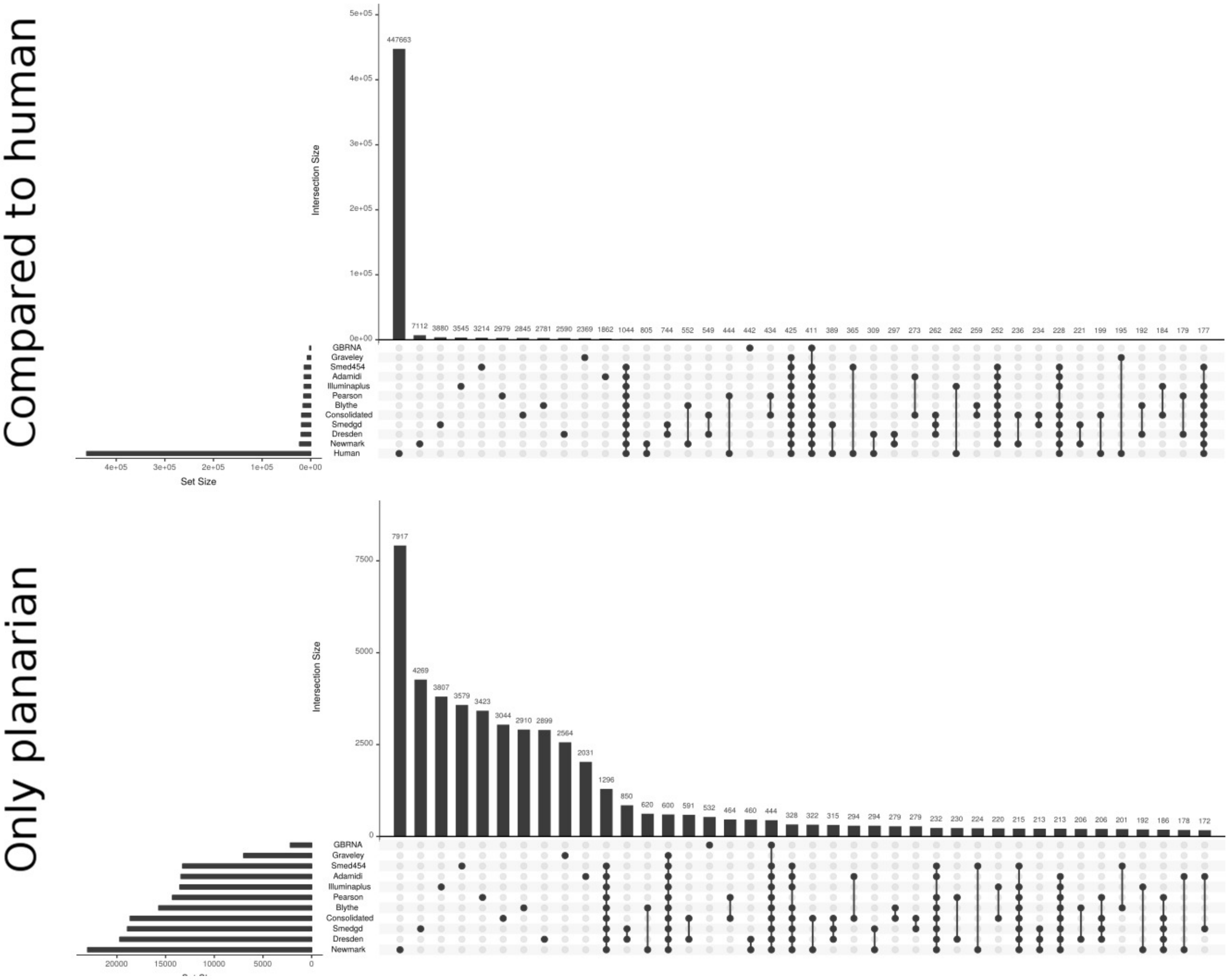

Supplementary Figure 5

Intersection of protein-protein interactions found in the reference interactome and the predicted planarian networks. For the predicted protein networks all the contig pairs were converted to their human homologous protein symbols before the intersections were made. The plots were created using the R package UpSetR.

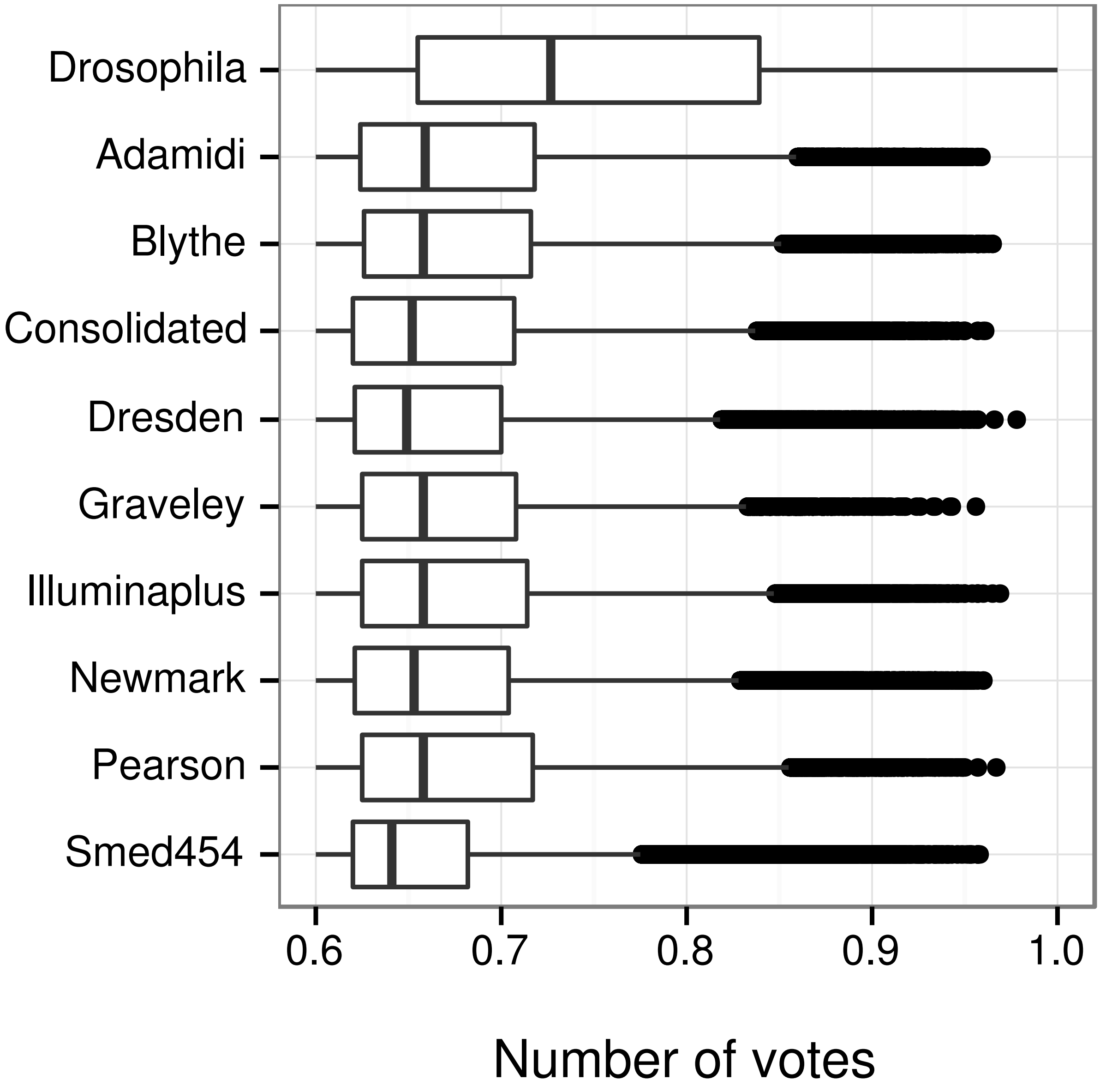

Supplementary Figure 6

Proportion of positive votes of random forest classification of protein-protein interactions per dataset. Note that the votes displayed for the training set correspond to the out of bag votes.

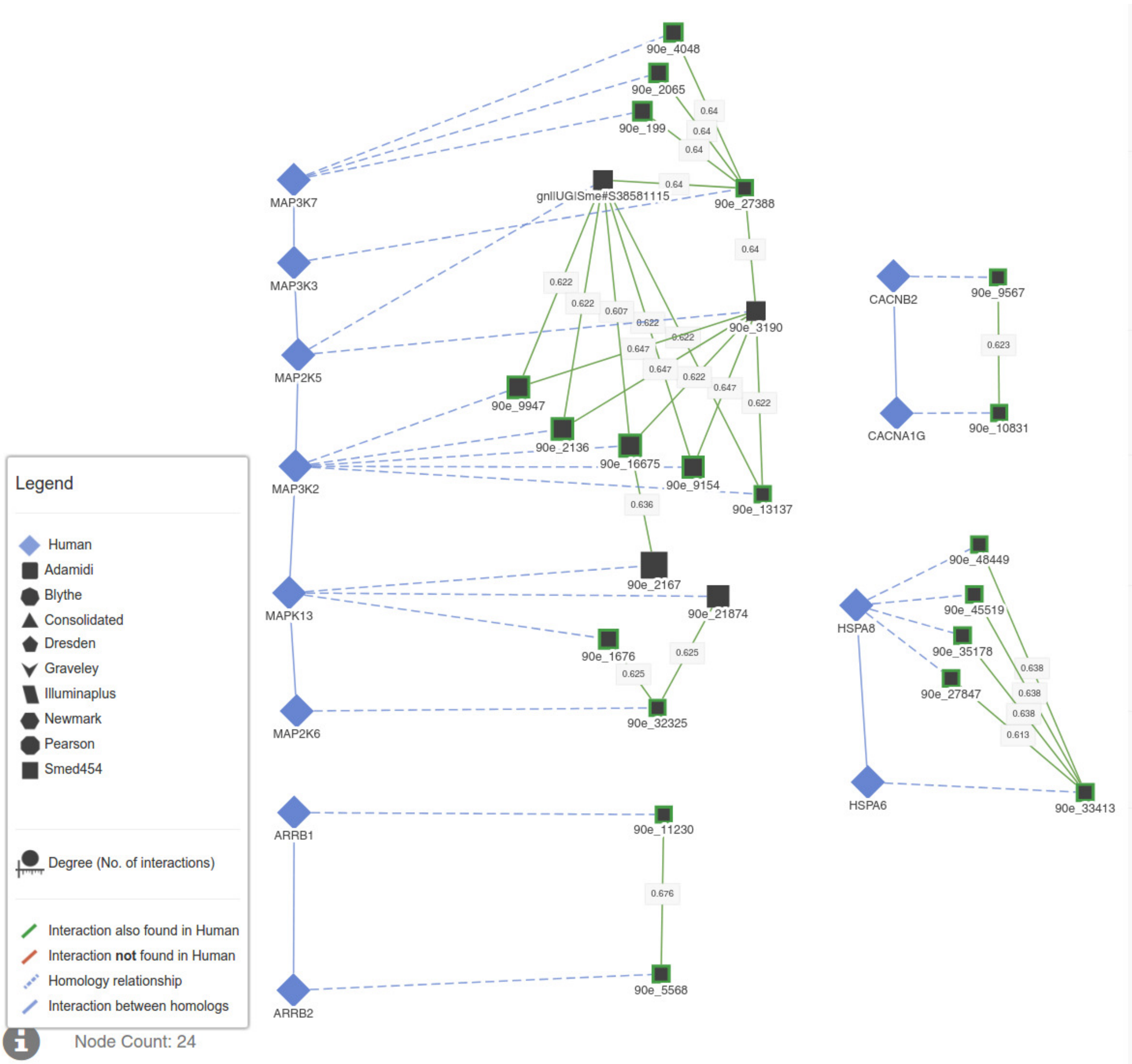

Supplementary Figure 7

Part of the MAPK signalling pathway (*KEGG:hsa04010*) projected over the *Smed454* transcriptome. All the proteins annotated to participate in the pathway were searched through the NetExplorer form, and then the retrieved nodes were filtered, removing all the interactions not found in human, and all the nodes that were not annotated in the Kegg pathway.

Supplementary Table 1

Description of the features used by the random forest classifier to predict protein-protein interactions.

FEATURE NAME	DESCRIPTION
PATH_LENGTH	Shortest path length between the homologous proteins in the reference human network.
CELLCOM_NTO	Cellular component Gene Ontology normalized term overlap between the human homologous proteins.
BIOPROC_NTO	Biological Process Gene Ontology normalized term overlap between the human homologous proteins.
NOG_EVAL_2	E-value of HMMER alignment between Open Reading Frame of planarian transcript 2 of possible interacting pair with its best EggNOG model.
MOLFUN_NTO	Molecular function Gene Ontology normalized term overlap between the human homologous proteins.
NOG_EVAL_1	E-value of HMMER alignment between Open Reading Frame of planarian transcript 1 of possible interacting pair with its best EggNOG model.
BLAST_EVAL_1	E-value of BLAST alignment between planarian transcript 1 of possible interacting pair with its best homologous human protein.
BLAST_EVAL_2	E-value of BLAST alignment between planarian transcript 2 of possible interacting pair with its best homologous human protein.
BLAST_COV_1	Query coverage of BLAST alignment between planarian transcript 1 of possible interacting pair with its best homologous human protein.
BLAST_COV_2	Query coverage of BLAST alignment between planarian transcript 2 of possible interacting pair with its best homologous human protein.
PFAM_SC_1	Meta-alignment score between Open Reading Frame of planarian transcript 1 and best human homologous protein.
PFAM_SC_2	Meta-alignment score between Open Reading Frame of planarian transcript 2 and best human homologous protein.
NOG_BRH_1	Boolean variable indicating if the Open Reading Frame of planarian transcript 1 of possible interacting pair is a best reciprocal hit of its assigned homologous human protein in the EggNOG alignment.
NOG_BRH_2	Boolean variable indicating if the Open Reading Frame of planarian transcript 2 of possible interacting pair is a best reciprocal hit of its assigned homologous human protein in the EggNOG alignment.
BLAST_BRH_1	Boolean variable indicating if the Open Reading Frame of planarian transcript 1 of possible interacting pair is a best reciprocal hit of its assigned homologous human protein in the BLAST alignment.
BLAST_BRH_2	Boolean variable indicating if the Open Reading Frame of planarian transcript 2 of possible interacting pair is a best reciprocal hit of its assigned homologous human protein in the BLAST alignment.
DOMAIN_INT_SCORE	Summatory of all the domains found in the Open Reading Frames of the planarian transcripts that are annotated as interacting in the 3did database
PFAM_BRH_1	Boolean variable indicating if the Open Reading Frame of planarian transcript 1 of possible interacting pair is a best reciprocal hit of its assigned homologous human protein in the PFAM meta-alignment.
PFAM_BRH_2	Boolean variable indicating if the Open Reading Frame of planarian transcript 2 of possible interacting pair is a best reciprocal hit of its assigned homologous human protein in the PFAM meta-alignment.

Supplementary Table 2

Results of the comparison between TransPipe and BIPS (Garcia-Garcia *et al.* 2012) to predict protein-protein interactions over a reference dataset of 1,707 *Caenorhabditis elegans* transcript sequences and 8,207 interactions downloaded from BioGRID. BIPS was configured to use the default settings and only Human sequences and interactions were selected as reference. Both tools have very similar performance measures on this dataset, with BIPS having a better precision and specificity and TransPipe having the best recall, accuracy, F-measure and Matthews correlation coefficient. The differences that can be seen between these performance measures and the Out Of Bag validation that we performed on our work can be due to several reasons: firstly, the set of available protein-protein interactions for *C. elegans* on BioGRID is very small (only 8,207), secondly, the proportion of negatives and positives on the set is even more unbalanced that the one of our *Drosophila melanogaster* training set (with even more negative cases in relation to positive pairs), and finally, the small number of sequences results in a very low number of *C. elegans* sequences having a human homolog.

**Tool**	**Precision**	**Recall**	**Specificity**	**Accuracy**	**F-measure**	**Matthews Coeff.**
**TransPipe**	0.0197	0.0599	0.9943	0.9926	0.0296	0.0301
**BIPS**	0.0565	0.0100	0.9968	0.9784	0.0170	0.0161
