# Supplementary material for: PlanNET: homology-based predicted interactome for multiple planarian transcriptomes
Source: Bioinformatics. 2017 Nov 24;34(6):1016–23. doi: 10.1093/bioinformatics/btx738 (PMC5860622; doi:10.1093/bioinformatics/btx738)
Supplement: Supplementary Data [file btx738_supp.zip › btx738-suppl_data/supplementary_table_1.pdf]

Supplementary Table 1

Description of the features used by the random forest classifier to predict protein-protein interactions.

FEATURE NAME	DESCRIPTION
PATH_LENGTH	Shortest path length between the homologous proteins in the reference human network.
CELLCOM_NTO	Cellular component Gene Ontology normalized term overlap between the human homologous proteins.
BIOPROC_NTO	Biological Process Gene Ontology normalized term overlap between the human homologous proteins.
NOG_EVAL_2	E-value of HMMER alignment between Open Reading Frame of planarian transcript 2 of possible interacting pair with its best EggNOG model.
MOLFUN_NTO	Molecular function Gene Ontology normalized term overlap between the human homologous proteins.
NOG_EVAL_1	E-value of HMMER alignment between Open Reading Frame of planarian transcript 1 of possible interacting pair with its best EggNOG model.
BLAST_EVAL_1	E-value of BLAST alignment between planarian transcript 1 of possible interacting pair with its best homologous human protein.
BLAST_EVAL_2	E-value of BLAST alignment between planarian transcript 2 of possible interacting pair with its best homologous human protein.
BLAST_COV_1	Query coverage of BLAST alignment between planarian transcript 1 of possible interacting pair with its best homologous human protein.
BLAST_COV_2	Query coverage of BLAST alignment between planarian transcript 2 of possible interacting pair with its best homologous human protein.
PFAM_SC_1	Meta-alignment score between Open Reading Frame of planarian transcript 1 and best human homologous protein.
PFAM_SC_2	Meta-alignment score between Open Reading Frame of planarian transcript 2 and best human homologous protein.
NOG_BRH_1	Boolean variable indicating if the Open Reading Frame of planarian transcript 1 of possible interacting pair is a best reciprocal hit of its assigned homologous human protein in the EggNOG alignment.
NOG_BRH_2	Boolean variable indicating if the Open Reading Frame of planarian transcript 2 of possible interacting pair is a best reciprocal hit of its assigned homologous human protein in the EggNOG alignment.
BLAST_BRH_1	Boolean variable indicating if the Open Reading Frame of planarian transcript 1 of possible interacting pair is a best reciprocal hit of its assigned homologous human protein in the BLAST alignment.
BLAST_BRH_2	Boolean variable indicating if the Open Reading Frame of planarian transcript 2 of possible interacting pair is a best reciprocal hit of its assigned homologous human protein in the BLAST alignment.
DOMAIN_INT_SCORE	Summatory of all the domains found in the Open Reading Frames of the planarian transcripts that are annotated as interacting in the 3did database
PFAM_BRH_1	Boolean variable indicating if the Open Reading Frame of planarian transcript 1 of possible interacting pair is a best reciprocal hit of its assigned homologous human protein in the PFAM meta-alignment.
PFAM_BRH_2	Boolean variable indicating if the Open Reading Frame of planarian transcript 2 of possible interacting pair is a best reciprocal hit of its assigned homologous human protein in the PFAM meta-alignment.
