# Supplementary material for: PlanNET: homology-based predicted interactome for multiple planarian transcriptomes
Source: Bioinformatics. 2017 Nov 24;34(6):1016–23. doi: 10.1093/bioinformatics/btx738 (PMC5860622; doi:10.1093/bioinformatics/btx738)
Supplement: Supplementary Data [file btx738_supp.zip › btx738-suppl_data/supplementary_table_2.pdf]

## Supplementary Table 2

Results of the comparison between TransPipe and BIPS (Garcia-Garcia *et al.* 2012) to predict protein-protein interactions over a reference dataset of 1,707 *Caenorhabditis elegans* transcript sequences and 8,207 interactions downloaded from BioGRID. BIPS was configured to use the default settings and only Human sequences and interactions were selected as reference. Both tools have very similar performance measures on this dataset, with BIPS having a better precision and specificity and TransPipe having the best recall, accuracy, F-measure and Matthews correlation coefficient. The differences that can be seen between these performance measures and the Out Of Bag validation that we performed on our work can be due to several reasons: firstly, the set of available protein-protein interactions for *C. elegans* on BioGRID is very small (only 8,207), secondly, the proportion of negatives and positives on the set is even more unbalanced that the one of our *Drosophila melanogaster* training set (with even more negative cases in relation to positive pairs), and finally, the small number of sequences results in a very low number of *C. elegans* sequences having a human homolog.

| **Tool** | **Precision** | **Recall** | **Specificity** | **Accuracy** | **F-measure** | **Matthews Coeff.** |
|---|---|---|---|---|---|---|
| **TransPipe** | 0.0197 | 0.0599 | 0.9943 | 0.9926 | 0.0296 | 0.0301 |
| **BIPS** | 0.0565 | 0.0100 | 0.9968 | 0.9784 | 0.0170 | 0.0161 |
